# Curcumin–Copper Complex Nanoparticles for the Management of Triple-Negative Breast Cancer

**DOI:** 10.3390/nano8110884

**Published:** 2018-11-01

**Authors:** Khaled Greish, Valeria Pittalà, Sebastien Taurin, Safa Taha, Fatemah Bahman, Aanchal Mathur, Anfal Jasim, Fatima Mohammed, Ibrahim M. El-Deeb, Salim Fredericks, Fiza Rashid-Doubell

**Affiliations:** 1 Department of Molecular Medicine, College of Medicine and Medical Sciences, and Nanomedicine Research Unite, Princess Al-Jawhara Centre for Molecular Medicine and Inherited Disorder, Arabian Gulf University, Manama 328, Bahrain; sebastient@agu.edu.bh (S.T.); safat@agu.edu.bh (S.T.); fato88.fb@gmail.com (F.B.); aanchalmathur96@gmail.com (A.M.); anfalaaj@agu.edu.bh (A.J.); 2 Department of Oncology, Suez Canal University, Ismailia 007, Egypt; 3 Department of Drug Sciences, University of Catania, Catania I-95125, Italy; vpittala@unict.it; 4 Department of Basic Medical Sciences, Royal College of Surgeons in Ireland (RCSI), Medical University of Bahrain, Busaiteen 221, Bahrain; Fatimahassan221@gmail.com (F.M.); imeldeeb@gmail.com (I.M.E.-D.); sfredericks@rcsi-mub.com (S.F.); frdoubell@rcsi-mub.com (F.R.-D.)

**Keywords:** Curcumin, curcumin complex, copper, nanoparticles, breast cancer, UV stability, release study, CD, TNBC

## Abstract

Breast cancer is the most common cancer diagnosed among females worldwide. Although breast cancer survival has largely improved in the past 30 years, it remains highly heterogeneous in its response to treatment. Triple-negative breast cancer (TNBC) is a subtype of breast cancer that lacks the expression of the estrogen receptor (ER), progesterone receptor (PR) and epidermal growth factor receptor-2 (Her2). While TNBC may initially be responsive to chemotherapy, recurrence and subsequent high mortality rates are frequently reported. Studies have shown curcumin and its derivatives to be effective against TNBC cell lines in vitro. To improve its anti-cancer effects, we have synthesized Fe^3+^–curcumin (Fe–Cur_3_) and Cu^2+^–curcumin (CD) complexes and investigated them experimentally. Further, CD was encapsulated into a poly(styrene)-co-maleic acid (SMA) micelle to enhance its stability. We assessed the cytotoxicity of these formulations both in vitro and in vivo. SMA–CD demonstrated dose-dependent cytotoxicity and abolished TNBC tumor growth in vivo. The encapsulation of the curcumin–copper complex improved its anti-cancer activity without overt adverse effects in a murine model of TNBC. These results provide evidence and insights into the value of nanoformulations in enhancing drug-delivery and increasing the potential therapeutic efficacy of curcumin derivatives.

## 1. Introduction

Breast cancer is the most common cancer diagnosed in women worldwide. Despite improvements in survival rates as screening and treatment improved, breast cancer remains the second biggest cause of cancer-related deaths in the USA [1]. Breast cancers are highly heterogeneous in their responses to treatments, and patient outcomes have proven to be difficult to predict [2]. There are 20 major types of breast cancers with multiple subtypes. Classification of these cancers are based on histopathology and are broadly stratified into five different molecular profiles according to gene expression patterns, these being: luminal A, luminal B, human epidermal growth factor receptor 2 (HER2)-enriched, basal-like, and normal-like breast cancer [3,4]. Therapeutic options and approaches are primarily governed by the expression and the relative amount of the three essential biomarkers: estrogen receptor-α (ER-α), progesterone receptor (PR), and HER2 [5]. Targeted therapies have drastically improved the survival of patients with hormone-responsive cancers. However, challenges remain regarding patients which breast tumors that do not express ER-α, PR, and HER2 in significant amounts. These are commonly referred to as triple-negative breast cancers (TNBC) and are aggressive tumors with a high recurrence rate and poor prognosis [6]. Hormonal therapies are not a satisfactory option for patients with these endocrine-resistant tumors. Chemotherapy, surgery and/or radiotherapy are currently the only treatments available for these patients and relapse and metastasis occur frequently within the first few years of diagnosis [7]. 

Because of these limited treatment options for TNBCs, research efforts have turned to investigation of novel compounds and naturally occurring substances. Recently, new potential therapies, drawn from the natural environment, have been assessed [8,9]. The phytocompound curcumin [(1*E*,6*E*)-1,7-bis(4-hydroxy-3-methoxyphenyl)-1,6-heptadiene-3,5-dione] is one such compound. It is a yellow compound extracted from the root and rhizome of *Curcuma longa* (Turmeric). Curcumin is the major component of Indian curry and has been traditionally used as a spice, a natural dye, and for traditional medicine. The effects of curcumin on biological systems are pleiotropic. It acts as an antioxidant, anti-inflammatory, and anti-cancer agent and appears to lack the side effects and toxicity associated with certain established drugs [10]. Curcumin has been shown to have selective toxicity for various cancer cell types. These include pancreatic, breast, prostate and lung cancers and involve multiple concurrent signaling pathways. [11,12]. However, curcumin’s transition into the clinic has been impaired by its low water solubility and poor stability, resulting in poor pharmacological bioavailability [13,14]. Structurally, curcumin is formed by two feruloyl moieties linked by a methylene branch. The precise mechanism through which curcumin undergoes degradation is not clear, but the conjugated α,β-unsaturated β-diketo residue seems to play a major role [15]. Various approaches have been adopted to overcome curcumin’s unfavorable pharmacokinetic profile [16], such as derivatization and encapsulation. Curcumin–metal complex formation involves the disappearance of the β-diketo moiety and renders the final adduct more stable and more cytotoxic to tumor cells [17,18]. Encapsulation of these complexes with poly(styrene)-co-maleic acid (SMA) micelles may promote their accumulation in cancer tissues, increase their water solubility, stability, and prolong their half-life in circulation. Here, we aim to address the issues of stability and delivery to target tissue in vivo by synthesizing metal complexes of curcumin (Cu^2+^ (CD) and with Fe^3+^ (Fe–Cur_3_)) followed by SMA micelle encapsulation of CD. These preparations were then tested both in vitro and in vivo. 

## 2. Results

### 2.1. Preparation and Characterization of CD Micelles

The SMA micelles were synthesized as reported in the experimental section. The SMA–CD had a recovery of 80%. The micellar system had a water solubility above 30 mg/mL. The loading was 18%, and the SMA–CD micelle had a mean diameter of 248 nm. The polydispersity index (PDI) of SMA–CD micelles was 0.274, and the zeta potential was −0.11 mV in deionized water (Table 1).

### 2.2. In Vitro SMA–CD Drug Release Studies

**Figure 1:**
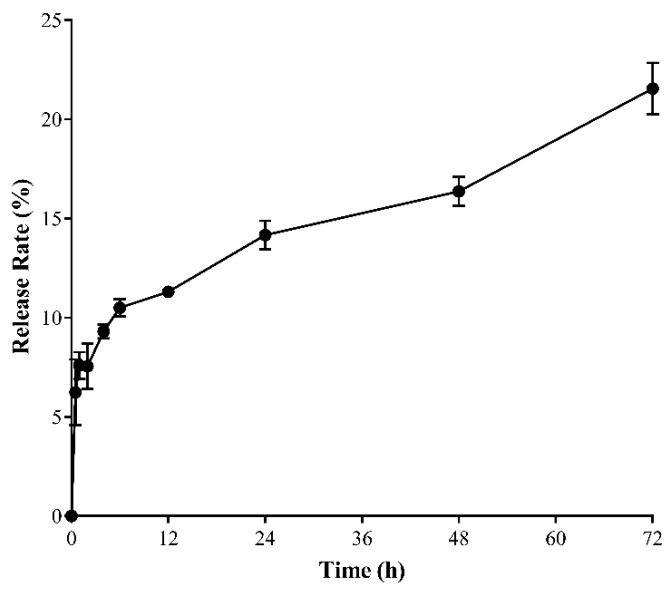
The release rate of CD from the SMA micelle. The release rate was determined using an in vitro dialysis system. Each point represents the mean ± standard deviation (SD) of triplicates. Data are presented as the average ± the standard error of the mean (SEM) (*n* = 3).

The release of the CD from SMA–CD was measured at physiological pH 7.4 for 72 h. As can be seen in Figure 1, approximately 20% of CD was released after 72 h at physiological pH. After 12 h, the cumulative release was 11%. From 12 h to 72 h, the release slowed down and was constant. The slow release reflects the stability of the micellar system in aqueous media. 

### 2.3. Drug Stability Studies of CD and SMA–-CD in Phosphate-Buffered Saline and PBS/Fetal Bovine Serum (FBS)

**Figure 2:**
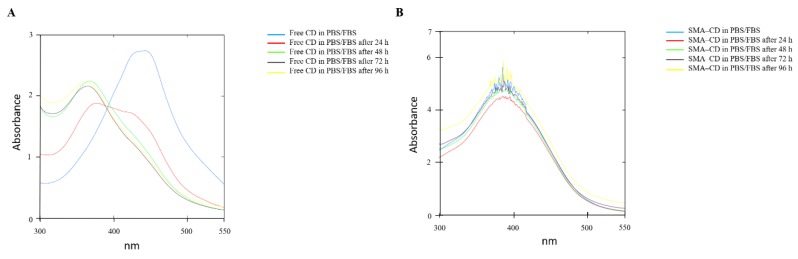
UV/Vis absorption spectra of CD and SMA–CD incubated at 37 °C in phosphate-buffered saline (PBS)/fetal bovine serum (FBS) (4:6) for 96 h. (**A**) Superimposing UV/Vis absorption spectra of CD measured at 0 h, 24 h, 48 h, 72 h, and 96 h; (**B**) Superimposing UV/Vis absorption spectra of SMA–CD measured at 0 h, 24 h, 48 h, 72 h, and 96 h. All experiments were performed in triplicate.

The stability of CD and SMA–CD in phosphate-buffered saline (PBS) (Figure S1) or PBS/fetal bovine serum (FBS) (4:6) at 37 °C, was assessed by measuring the UV/Vis absorption spectra (250 nm to 550 nm) after 24 h and for a period of 48 h or 96 h (Figure 2). As reported in Figure S1A, CD after 24 h in PBS demonstrated a slight shift towards a lower wavelength and different UV absorption spectra. In PBS/FBS, the CD UV spectra significantly changed (Figure 2A). On the other hand, SMA–CD showed the same UV spectra over the whole experiment (Figure S1B and Figure 2B). The change in the UV spectra of CD indicates lower stability when compared to the micellar system, both in PBS and in PBS/FBS.

### 2.4. CD Interaction with Plasma Proteins

CD was incubated at 37 °C for 30 min in FBS/PBS (4:6) at pH 7.4 to determine the interaction of the free curcumin complex with plasma proteins. The size distribution of the sample containing CD changed very fast for the control, suggesting the presence of complex interactions between CD and plasma proteins (Figure S2).

### 2.5. Cytotoxic Effect of Fe–Cur_3_, CD, and SMA–CD on Breast Cancer Cells

**Figure 3:**
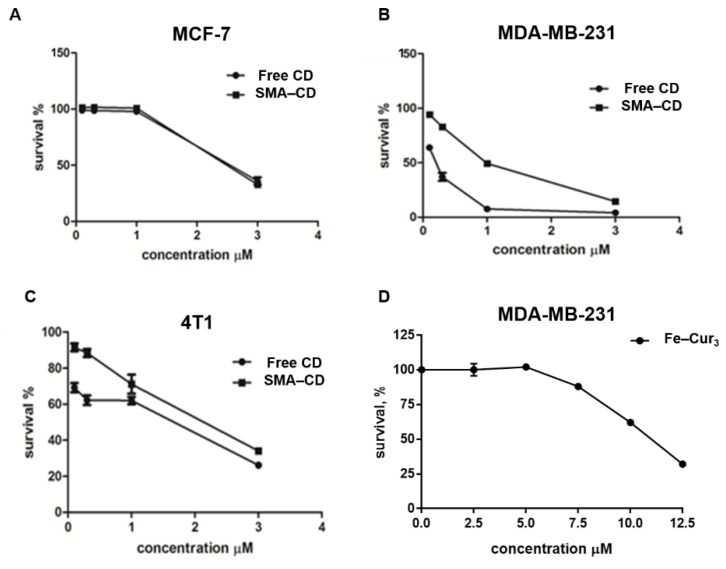
In vitro cytotoxic effect of CD, SMA–CD and Fe–Cur_3_, on hormone-responsive (MCF-7) and hormone resistant breast cancer cells (MDA-MB-231 and 4T1). (**A**) MCF-7, (**B**) MDA-MB-231, and (**C**) 4T1 cells were treated with different concentrations of CD and SMA–CD. (**D**) MDA-MB-231 cells were treated with Fe–Cur_3_. All cells were incubated for 48 h at 37 °C, 5% CO_2_. Control wells were treated with vehicle only (0.2% dimethyl sulfoxide (DMSO)). At the end of treatment, the cell number was determined using the sulforhodamine B assay. Data are expressed as mean ± SEM (*n* = 3). Nonlinear regression and IC_50_ value determination was performed using GraphPad Prism 6. Differences were considered significant at *p* < 0.05.

**Figure 4:**
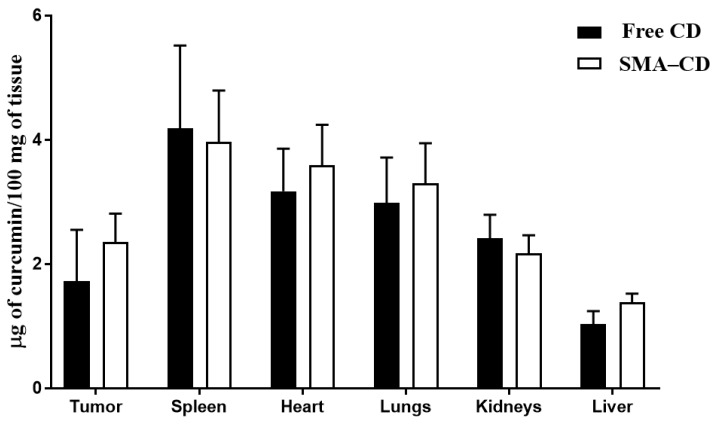
Quantitative determination of CD amount (µg) per 100 mg of tissue. Tissue distribution of CD at 24 h following intravenous injection of CD and SMA–CD to Balb/c mice bearing 4T1 tumors (*n* = 6). Data are presented as the average ± standard error.

The cytotoxicity of Fe–Cur_3_, CD, and SMA–CD on cell viability was assessed using three breast cancer cell lines, namely human MCF-7 cells, MDA-MB-231 cells, and murine 4T1 cells (Figure 3). The effect of the different preparations on cell viability was expressed as the concentration sufficient to kill 50% of the cells (IC_50_) as outlined in Table 2. Treatment of TNBC cells (MDA-MB-231 and 4T1, Figure 4B,C) showed a higher sensitivity to CD formulations. In both cell lines, CD showed a slightly enhanced cytotoxicity with respect to SMA–CD (MDA-MB-231 cells IC_50_ = 0.125 µM and 0.892 µM, respectively; 4T1 cells IC_50_ = 0.892 µM and 1.935 µM, respectively). This result is consistent with the lower in vitro activity of nanocarriers [19]. Micelles are internalized into the cells via endocytosis [20], a time and energy-dependent process in contrast to the simple diffusion of the free complex. MCF-7 cells (Figure 3A) appeared more resistant to the cytotoxic effect of the curcumin complexes and micelles when compared to MDA-MB-231 and 4T1 cells and showed that both CD and SMA–CD had no significant difference in their cytotoxic profile after 48 h incubation (Table 2, IC_50_ of 3.373 µM and 3.370 µM, respectively). 

### 2.6. Maximum Tolerated Dose (MTD)

To evaluate the safety of CD and SMA–CD, a maximum tolerated dose (MTD) study was performed following the recommendation of the Organization for Economic Co-operation and Development (OECD) test 420 [21]. The acute toxicity MTD for SMA–CD following intravenous dose escalation was found to be 120 mg/kg and that of the free CD to be 50 mg/kg.

### 2.7. Biodistribution of CD and SMA–CD

Balb/c mice bearing 4T1 mammary carcinoma tumors were injected with CD and SMA–CD (10 mg/kg and 20 mg/kg) to examine the dose response. CD was measured in tissues 24 h following injection. As reported in Figure 4, the curcumin complex was distributed in the lungs, spleen, liver, heart, kidneys, and tumors. There was a slightly greater (but not significant) accumulation of CD in the tumor and liver following the injection of SMA–CD when compared to the free complex injection. In the tumor and liver, the content of CD expressed as µg/mg of tissue was 1.37- and 1.33-fold higher, respectively, when comparing SMA–CD to free CD injection (Figure 4); however, this difference was not statistically significant. No difference was observed in the kidney, lung, heart, and spleen (Figure 4). The higher amount of CD measured in the liver following SMA–CD injection may be due to high blood perfusion, large fenestration size, and the presence of reticuloendothelial cells in the liver.

### 2.8. Effect of CD and SMA–CD on 4T1 Tumor Growth

**Figure 5:**
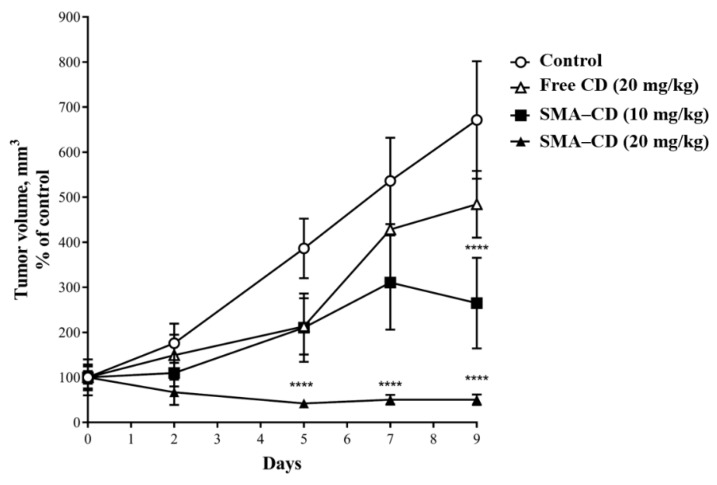
In vivo anti-tumor effect of CD and SMA–CD on 4T1 tumor-bearing Balb/c mice. Mice were treated for ten days with either CD 20 mg/kg and SMA–CD 10 mg/kg and 20 mg/kg. The control group was injected with a physiological saline solution. Tumor growth/size was estimated manually using a caliper. Data are presented as the average tumor volume ± SEM. **** *p* < 0.0001. After ten days of treatment, the treatment with free CD reduced tumor growth by 28%, while SMA–CD (10 mg/kg) decreased the tumor volume by 61% and SMA–CD (20 mg/kg) abolished tumor growth.

The anti-tumor effect of CD and SMA–CD was evaluated using Balb/c mice harboring 4T1 tumors over a treatment period of ten days. As shown in Figure 5, tumor growth was initially delayed upon treatment with free CD (20 mg/kg), but ultimately the tumor size was not statistically different from the control group after ten days. Treatment with SMA–CD appeared to be dose-dependent and, as seen in Figure 5, the encapsulation of CD increased its efficacy significantly in vivo. A single dose of SMA–CD at 10 mg/kg reduced the tumor growth by 2.1-fold while a dose of 20 mg/kg completely stopped the tumor growth for the duration of the study (Figure 5). The therapeutic efficacy of CD and SMA–CD treatments were not associated with any significant weight loss during the treatment period. 

## 3. Discussion

Breast cancers are disparate in their response to treatments, with TNBC lacking targeting therapies and remaining one of the most aggressive cancers characterized by a high recurrence rate. The need for targeted therapy for the treatment of TNBC has led to the search for new therapies. Phytocompounds are being increasingly acknowledged as a useful complement to standard anti-cancer therapy. Curcumin is a natural polyphenol compound that exerts anti-cancer effects via multiple signaling pathways. Its pleiotropy is probably due to the presence of several reactive functional groups in its structure. Despite the safety of curcumin at high doses, its clinical use has been hampered by significant limitations, such as a scarce ADME (absorption, distribution, metabolism, and excretion) profile and poor chemical stability.

We have demonstrated the effects of two curcumin complexes (copper and iron) on breast cancer cells in vitro using MCF-7, MDA-MB-231, and 4T1 cell lines. The cell viability assay (Table 2) demonstrated higher cytotoxicity of the copper–curcumin complex relative to the cytotoxicity observed with the iron–curcumin complex. The purpose of synthesizing and testing these metal complexes was to address the contribution of coordination metals to the overall activity of curcumin derivatives. The CD was cytotoxic in the sub-micromolar range against human and murine TNBC cells (MDA-MB-231 IC_50_ = 0.125 µM; 4T1 IC_50_ = 0.892 µM, respectively). Pure non-complexed curcumin has previously been shown to have a higher IC_50_ value (25.6 µM) in MDA-MB-231 cells [22]. Curcumin complex formation with a metal involves binding through the β-diketo function which renders the derivative more stable and more cytotoxic than the parent compound [23]. 

Previous studies have shown copper alone to be cytotoxic against cancer cells, albeit at a concentration above 100 µM [24], although copper nanoparticles coated with albumin displayed cytotoxicity in TNBC cells above 50 µM [25]. Furthermore, a previous study using curcumin-capped copper nanoparticles failed to demonstrate any cytotoxicity against TNBC cell lines [26]. However, more recently, copper(II) phosphorus dendrimers demonstrated cytotoxicity against cancer cells [27].

Based upon our cytotoxicity data, the copper–curcumin complex was selected for further in vitro investigation. This was the more potent of the two complexes. However, the CD is poorly soluble in water. This poor solubility significantly influences all pharmacokinetic parameters in vitro, including bioavailability. We, therefore, attempted to increase the bioavailability of CD to improve drug delivery for in vivo evaluation. In an attempt to enhance CD accumulation at the tumor site and reduce off-target effects, the CD was encapsulated into SMA nanoparticles (NPs). SMA is an amphiphilic polymer that demonstrates significant biocompatibility. It is biodegradable and can bind non-covalently to plasma albumin. Importantly, it also has an increased potential to accumulate in tumors through enhanced permeability and retention effects (EPR) [28]. The slow release rate of CD from the NPs demonstrated its appropriateness for the in vivo application of drug delivery systems. SMA–CD showed a similar cytotoxicity profile as the free complex CD in MCF-7 cells. However, a decreased cytotoxicity was observed for the micelle formulation in the TNBC cell lines (MDA-MB-231 and 4T1 cells). This result is in agreement with previous reports demonstrating reduced pharmacological activity in vitro when delivered by NPs as compared to free drug delivery [19]. The micelles enter the cells through endocytosis in a time and energy-dependent process opposing the diffusion of most of the free drugs. 

The biodistribution of CD and SMA–CD were evaluated using the 4T1 tumor murine model of TNBC. The period of 24 h was chosen as the EPR effect is observed after 6 h, reaches a peak after 24 h and keeps its plateau from 24 h to 72 h [29,30,31,32]. The biodistribution of the free complex and the encapsulated formulation was comparable in all organs, including the tumors. Given the negligible differences in biodistribution between CD and SMA–CD, it was not possible to rely on the EPR effect to explain the remarkable differences in the anti-tumor activity between both formulations. A single injection of SMA–CD at the dose of 10 mg/kg was able to reduce the tumor growth by approximately 61% after ten days, while SMA–CD at the dose of 20 mg/kg stabilized the tumor size for ten days. CD at 20 mg/kg demonstrated tumor control comparable to SMA–CD 10 mg/kg until day 5. However, the tumor regrew with a reduction in the tumor size of 28% at the end of the treatment period. No significant signs of toxicity were associated with the treatments. We, therefore, checked the compound’s stability. The UV/Vis spectra of CD changed significantly upon incubation in PBS/FBS when compared to SMA–CD, demonstrating that the micellar formulation protected CD from degradation in the serum.

Plasma protein binding of free CD can render it into a nanoparticle size that is similar to the SMA encapsulated curcumin used here, thus allowing it to take advantages of the EPR effect. The interaction of curcumin with plasma proteins in vivo has been previously demonstrated [33]. Our CD preparation, upon incubation with plasma proteins, showed changes in the size distribution of the sample, suggesting interactions between CD and FBS proteins. Our data demonstrate the value of protecting curcumin derivatives from degradation in the plasma. Nanocarrier systems, such as nanomicelles in our case, can achieve this protection. In contrast to our initial hypothesis which related to improving CD concentration at the tumor site through the EPR effect connected to nanosized formulations, CD itself (by binding to plasma proteins) acts as a self-assembled nanosystem with the same favorable tumor accumulations. The main advantage of SMA–CD would thus rely on preserving the activity of the parent compound. In light of this explanation, further measures need to be taken to improve curcumin-related research in the biomedical field. 

The free copper–curcumin complex and the SMA encapsulated complex showed significant cytotoxic effects. Both formulations showed cytotoxicity values in the lower micromolar or sub-micromolar concentration range in vitro. SMA–CD reduced tumor growth in a dose-dependent manner more efficiently than the non-encapsulated CD in vivo. Remarkably, at the highest dose, SMA–CD abolished murine 4T1 tumor growth. This effect may be attributed to the higher stability of the micellar system in biological fluids, independent of its increased ability to enter and accumulate in tumor cells. Taken together, this improved chemical stability and improved targeting of tumor cells may open up the way for new and enervative treatments for TNBC.

## 4. Materials and Methods 

Roswell Park Memorial Institute medium (RPMI) 1640 medium, fetal bovine serum (FBS), and TrypLE Express were purchased from ThermoFisher (ThermoFisher Scientific, Franklin, MA, USA). Curcumin, CuCl_2_, FeCl_3_, poly(styrene-co-maleic anhydride), cumene terminated (SMA) MW 1600, dioctadecyl3,3,30,30-tetramethylindocarbocyanine perchlorate (DiI), *N*-(3-dimethylaminopropyl)-*N*-ethylcarbodiimide hydrochloride (EDAC), l-glutamine, penicillin, streptomycin, sulforhodamine B (SRB) and tris(hydroxymethyl)aminomethane (TRIS) were purchased from Sigma-Aldrich Ltd. (St. Louis, MO, USA). Trichloroacetic acid (TCA) was secured from Global Science (Auckland, New Zealand). All solvents were reagent grade and were purchased from commercial vendors.

### 4.1. Synthesis of Fe–Cur_3_

Curcumin (2.21 g, 6 mmol) was dissolved in 50 mL of a mixture of methanol/dichloromethane (1:1), and an aqueous solution (5 mL) of FeCl_3_ (0.541 g, 2 mmol) was added. The mixture was refluxed for 3 h. The resulting solution was concentrated to a final volume of 10 mL and allowed to stand at room temperature. The precipitated complex was filtered off before being washed, first with cold water and then methanol. The crude was recrystallized from MeOH/dichloromethane (1:1). Finally, the synthesized complex was dried, dissolved in 30 mL of light petrol and 10 mL of ethyl acetate and left standing. The resulting precipitate was filtered under gravity, washed with light petrol (20 mL × 2), and dried to give the pure complex. 

### 4.2. Synthesis of CD

Curcumin complex with copper (CD) was obtained following the procedure reported by Barik et al. [34]. In this report an adaptation of the original procedure published by Dutta et al. [35] was followed. Curcumin (1.47 g, 4 mmol) was dissolved in methanol (100 mL) and an aqueous solution (10 mL) of CuCl_2_ (0.268 g, 2 mmol) was added. The mixture was refluxed for 3 h. The resulting solution was concentrated to a final volume of 20 mL and allowed to stand at room temperature. The precipitated complex was filtered off before being washed, first with cold water and then ethanol. The crude was recrystallized from MeOH/dichloromethane (1:1). Finally, the synthesized complex was dried, dissolved in 30 mL of light petrol and 10 mL of ethyl acetate and left standing. The resulting precipitate was filtered under gravity, washed with light petrol (20 mL × 2), and dried to give the pure complex as a yellow powder. 

### 4.3. Preparation and Characterization of SMA–CD Micelles

SMA micelles were prepared as described previously [36]. Briefly, SMA was hydrolyzed by dissolving the SMA in 1 M NaOH at 70 °C to achieve a concentration of 10 mg/mL. Hydrolyzed SMA was then adjusted to pH 5, and EDAC was added in a 1:1 ratio by weight to SMA. A solution of CD, dissolved in DMSO, was added to the SMA solution and the pH was maintained at 5 until it stabilized. Then, the pH was raised and maintained stably at 11, and finally lowered to 7.4. The solution was filtered four times using a Millipore Lab scale TFF (tangential flow filtration) system (Merck Millipore, Burlington, MA, USA) with a Pellicon XL 10 kDa cut-off membrane (EMD Millipore, Burlington, MA, USA). The solution was frozen at −80 °C overnight before being lyophilized to obtain the SMA–CD micelle powder.

#### 4.3.1. Loading of the SMA–CD Micelles

A standard curve of CD free drug was pre-prepared in DMSO and measured at 428 nm to determine the weight ratio of the loaded drug. The drug content of SMA–CD was determined by solubilizing SMA–CD (1 mg/mL) in DMSO and measuring the absorbance at 428 nm in comparison with the standard curve. Measurements from three independent experiments were conducted in triplicate. The loading was expressed as weight % of CD in the final micelle compared to the total weight of recovered SMA–CD. The SMA–CD loading was determined as 18%.

#### 4.3.2. Size, PDI, and Zeta Potential Determination of SMA–CD Micelles

Lyophilized SMA–CD micelles (1.5 mg/mL) were solubilized either in NaHCO_3_ (0.1 M, pH 7.8) to determine the size or distilled water to estimate the charge. All measurements for size distribution and zeta potential were carried out using the Malvern ZEN3600 (Malvern Instruments Inc., Westborough, MA, USA). Measurements from three independent experiments were conducted in triplicate.

#### 4.3.3. Drug Release Studies of SMA–CD in Distilled Water

The release of the CD from the micelle construct was evaluated using a dialysis method. SMA–CD micelles were solubilized at a concentration of 1 mg/mL in distilled water Using a dialysis bag with a 12 kDa molecular weight cutoff, 2 mL was dialyzed against 20 mL of distilled water (pH adjusted to 7.4). For up to 72 h, 1 mL of the sample inside the dialysis bag was removed, and the absorbance was measured at 428 nm using a UV spectrophotometer (UV-2600, Shimadzu, Japan) with a quartz cell of 1 cm path length, then returned to the dialysis bag. The % reduction of maximum absorbance at 428 nm from the 0-time point was recorded to signify the release rate. The choice of measuring the release rate from the inside sample is related to instability and poor water solubility of the free CD as described further in Section 2.3. All experiments were performed in triplicate. Percentage release is reported as mean percentage ± SEM.

#### 4.3.4. Drug Stability Studies of CD and SMA–CD in PBS or PBS/FBS

To test the stability of CD and SMA–CD, UV/Vis absorption spectra of CD and SMA–CD (0.01 mM) incubated at 37 °C in PBS or PBS/FBS (4:6) were measured over the wavelength range of 250–550 nm. The stability of the free complex and the micelle was monitored for 48 h or 96 h by observing the relative change in the absorption peak upon incubation of the compounds (0.01 mM) in the dark at 37 °C in a solution of PBS/FBS (6:4) or PBS. All experiments were performed in triplicate.

#### 4.3.5. CD Interaction with Plasma Proteins

CD complex (2 mg) was solubilized in 500 µL of methanol, and an aliquot of 20 µL was added to 980 µL of medium containing FBS/PBS (4:6), pH 7.4. The sample was incubated at 37 °C for 30 min. After this time, to determine the size, measurements were done using the Malvern ZEN3600 Zetasizer Nano series (Malvern Instruments, Malvern, UK) (Figure S1, Supporting Information). The results were obtained from three independent experiments and were compared to size measurements of medium containing FBS/PBS, pH 7.4.

### 4.4. In Vitro Studies

#### 4.4.1. Cell Culture

MDA-MB-231 (ATCC^®^ HTB-26™), 4T1 (ATCC^®^ CRL-2539), and MCF-7 (ATCC^®^ HTB22™) cells were obtained from the American Type Culture Collection (ATCC) (Manassas, VA, USA). MDA-MB-231 and MCF-7 cells were grown in DMEM/Ham’s F12 supplemented with 5% FBS, 2 mM l-glutamine, 100 µg/mL streptomycin, 100 U/mL penicillin and 2.2 g/L NaHCO_3_; 4T1 cells were grown in RPMI-1640 medium supplemented with 10% (v/v) fetal calf serum, 2 mM l-glutamine, 100 U/mL penicillin, and 100 µg/mL streptomycin. For all procedures, cells were harvested using TrypLE Express. All the cells were incubated at 37 °C in a humidified atmosphere with 5% CO_2_.

#### 4.4.2. Cell Viability

MDA-MB-231 (1 × 10^4^ cells/well), MCF-7 (1 × 10^4^ cells/well) and 4T1 cells (1 × 10^4^ cells/well) were seeded in 96-well plates, incubated for 24 h and then treated with a concentration range from 0.1 µM to 3 µM of either CD dissolved in 5% DMSO or SMA–CD dissolved in water. In all experiments, the cells were incubated for 48 h and then fixed using 10% trichloroacetic acid (TCA). Cell number was determined using the sulforhodamine B (SRB) assay as previously described [37]. The concentration required to decrease cell numbers by 50% (IC_50_) was determined by nonlinear regression using GraphPad Prism 7. Three independent experiments were performed in triplicate.

### 4.5. In Vivo Studies

Female Balb/c mice (6 weeks old, mean weight = 22 g) were supplied by the Laboratory Animal Care Facility (LACF) of the Arabian Gulf University (AGU), Bahrain. All animals were kept under standard conditions including a controlled temperature of 25 °C, a 12 h light–dark cycle, and had ad libitum access to food and water. Experiments were performed according to the roles and regulations of the AGU Animal Care Policy. CD was dissolved in DMSO and diluted in physiological saline to a 5% final percentage of DMSO and SMA–CD was dissolved in physiological saline.

#### 4.5.1. Maximum Tolerated Dose (MTD)

MTD testing was conducted according to OECD test 420 [21]. Briefly, nulliparous and non-pregnant female Balb/c mice (aged 6 weeks old) were treated with escalating doses of CD or SMA–CD micelles through the tail vein (*n* = 6 per group). Mice were initially dosed with CD 10 mg/kg and a SMA–CD equivalent dose of 10 mg/kg and body weight was measured daily. The dose was escalated daily until the maximum solubility of drugs was reached or body weight loss of >10% was observed.

#### 4.5.2. Biodistribution of CD and SMA–CD

Female Balb/c mice were implanted with 4T1 cell tumors (1 × 10^6^ cells) bilaterally on the flanks. When tumors reached 100 mm^3^, mice were randomly distributed into two groups (3 animals per group). The animals were injected with either CD or its equivalence in SMA–CD at 50 mg/kg via the tail vein. Mice were euthanized 24 h after the treatment and organs were collected. Internal organs (heart, lungs, liver, spleen, and kidneys) and tumor tissue were analyzed for CD content. SMA–CD was extracted using the methodology previously described [38]. Briefly, tissues were minced, weighed and snapped frozen before being pulverized. Frozen tissue powder (1 mg) was re-suspended in 67% ethanol and 4 M HCl (1 mL). The suspension was incubated at 70 °C for 0.5 h, sonicated and centrifuged to extract CD from tissue samples. CD content was determined by absorbance at 428 nm and compared to a CD calibration curve. CD content was normalized to the weight of tissue and to the total weight of the organs from which it was extracted.

#### 4.5.3. Effect of CD and SMA–CD on 4T1 Tumor Growth

Female Balb/c mice were injected with 4T1 mammary carcinoma cells (1 × 10^6^) bilaterally on the flanks. When tumors reached 100 mm^3^, mice were randomly distributed into five groups (control, CD 10 mg/kg, CD 20 mg/kg, SMA–CD 10 mg/kg, and SMA–CD 20 mg/kg; 5 animals per group). CD and SMA–CD were administered via tail vein on day 0. The control animals were injected with physiological saline. The day of first administration was set as day 0. Tumor volume was measured by manual caliper; the volume was estimated using the formula: $V_{tumor}=\frac{W^{2}\times L}{2}$, where *W* is the transverse section and *L* is longitudinal cross section. Tumor volumes were normalized to the initial tumor volume at the initiation of treatment (day 0) and represented as the mean ± SEM. The body weight of the mice was measured biweekly and normalized daily for ten days as an index of acute toxicity.

### 4.6. Statistical Analysis

In vitro experiments, such as the cytotoxicity assay, were routinely repeated at least three times. The statistical analysis was performed using the one-way analysis of variance (ANOVA). For in vivo experiments, statistical analysis was performed using the two-way ANOVA. All analyses were performed using the GraphPad Prism 7 Software. * *p* < 0.05 was considered statistically significant.

## Figures and Tables

**Table 1:** Characterization of poly(styrene)-co-maleic acid–(Cu^2+^–curcumin) (SMA–CD) complexes.

Micelle	Loading (*wt*/*wt*)	Size (nm)	PDI	Entrapment Efficiency (% EE)	Zeta Potential (mV)
SMA–CD	18%	248 ± 68	0.274	80%	−0.11 ± 0.03

Values are the mean of triplicate experiments. PDI = polydispersity index.

**Table 2:** The concentration sufficient to kill 50% of the cells (IC_50_) values for CD and SMA–CD on hormone-responsive (MCF-7 cells) and hormone resistant (MDA-MB-231 and 4T1 cells) breast cancer cells.

Compound	IC_50_ (µM) ^1^		
	MCF-7	MDA-MB-231	4T1
Fe–Cur_3_	>10	>10	>10
CD	3.37 ± 1.2	0.13 ± 0.07	0.89 ± 0.09
SMA–CD	3.37 ± 1.03	0.69 ± 0.13	1.94 ± 0.12

^1^ Data are shown as IC_50_ values in µM ± SD. Values are the mean of triplicate experiments.

## Supplementary Materials

The following are available online at http://www.mdpi.com/2079-4991/8/11/884/s1, Figure S1: UV/Vis absorption spectra of CD and SMA–CD incubated at 37 °C in PBS 48 h. (A) Superimposing UV/Vis absorption spectra of CD measured in PBS at 0, 24, and 48 h; (B) Superimposing UV/Vis absorption spectra of SMA–CD measured in PBS at 0, 24, and 48 h. All experiments were performed in triplicate, Figure S2: CD interaction with plasma proteins. The size of the CD complex with plasma proteins was determined by dynamic light scattering using the using the Malvern ZEN3600 Zetasizer Nano series. The Results were obtained from three independent experiments.
