# Supplementary material for: Curcumin–Copper Complex Nanoparticles for the Management of Triple-Negative Breast Cancer
Source: Nanomaterials (Basel). 2018 Nov 1;8(11):884. doi: 10.3390/nano8110884 (PMC6267006; doi:10.3390/nano8110884)
Supplement: Supplementary file 1 [file nanomaterials-08-00884-s001.pdf]

nanomaterials

MDPI

Article

Curcumin-Copper Complex Nanoparticles for the Management of Triple-Negative Breast Cancer.

Khaled Greish [1,*], Valeria Pittalà [2], Sebastien Taurin [1], Safa Taha[1], Fatemah Bahman[1], Aanchal Mathur[1], Anfal Jasim[1], Fatima Mohammed[3], Ibrahim M El-Deeb[3], Salim Fredericks[3], and Fiza Rashid-Doubell[3]

[1] Department of Molecular Medicine, College of Medicine and Medical Sciences, and Nanomedicine research unite, Princess Al-Jawhara Centre for Molecular Medicine and Inherited Disorder, Arabian Gulf University, Manama, Kingdom of Bahrain

[2] Department of Drug Sciences, University of Catania, Catania, Italy

[3] Department of Basic Medical Sciences, Royal College of Surgeons in Ireland (RCSI), Medical University of Bahrain, Bahrain

* Correspondence: khaledfg@agu.edu.bh; Tel.: +973 34358697

Received: 28 September 2018; Accepted: 25 October 2018; Published: 1 November 2018

Table of contents

CD interaction with plasma proteins (Figure S1) S1

UV/Vis absorption spectra of CD and SMA–CD incubated at 37 °C in PBS 48 h (Figure S2) S2.

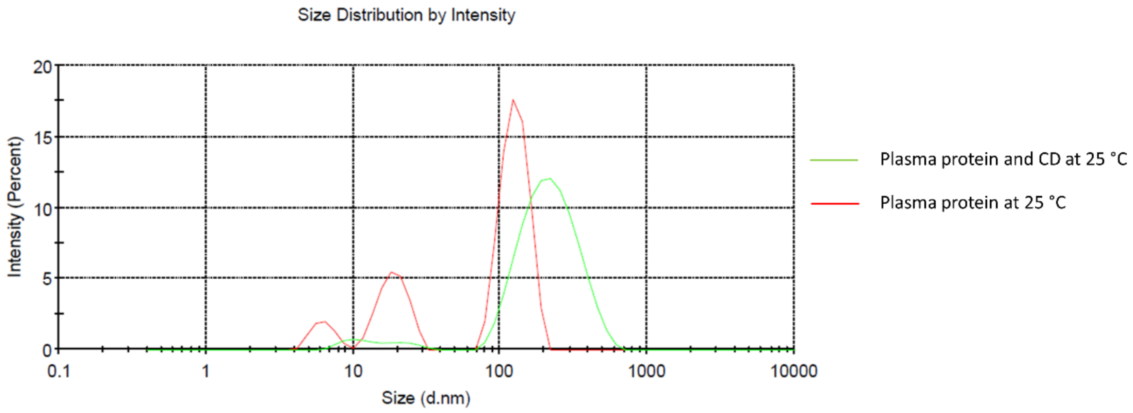

Figure S1. CD interaction with plasma proteins. The size of the CD complex with plasma proteins was determined by dynamic light scattering using the using the Malvern ZEN3600 Zetasizer Nano series. The Results were obtained from three independent experiments.

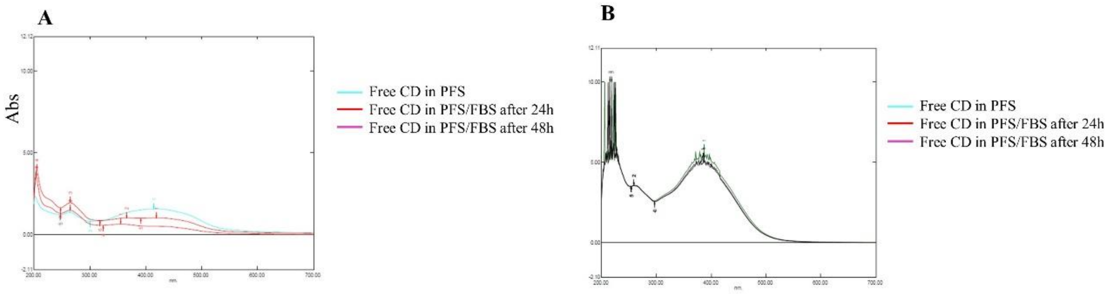

Figure S2. UV/Vis absorption spectra of CD and SMA–CD incubated at 37 °C in PBS 48 h. (**A**) Superimposing UV/Vis absorption spectra of CD measured in PBS at 0, 24, and 48 h; (**B**) Superimposing UV/Vis absorption spectra of SMA–CD measured in PBS at 0, 24, and 48 h. All experiments were performed in triplicate.

Sample Availability: Samples of the compounds ...... are available from the authors.

© 2018 by the authors. Licensee MDPI, Basel, Switzerland. This article is an open access article distributed under the terms and conditions of the Creative Commons Attribution (CC BY) license (http://creativecommons.org/licenses/by/4.0/).
